# Effects of talker gender and face masks on the speech recognition of 6-year-old children in a classroom

**DOI:** 10.3389/fpubh.2025.1430530

**Published:** 2025-06-16

**Authors:** Miji Kwon, Wonyoung Yang

**Affiliations:** ^1^Department of Speech-Language Pathology, College of Health Welfare, Gwangju University, Gwangju, Republic of Korea; ^2^Division of Architecture, College of Engineering, Gwangju University, Gwangju, Republic of Korea

**Keywords:** face masks, speech recognition, 6-year-old children, talker gender, monosyllabic word list, reverberation time, noise, classroom acoustics

## Abstract

Although mandatory wearing of face masks for 3 years owing to COVID-19 might have strongly affected children’s language development, its effects on their speech recognition based on the talker’s gender remain unknown. This study examined how face mask usage affects children’s speech recognition, focusing on the interaction between the talker’s gender and the child listener’s characteristics under realistic acoustic conditions with room reverberation and background noise. Speech recognition was assessed in 43 6-year-old children who had worn masks for two or more years during preschool. Auralisation techniques using male and female professional voice actors’ recordings under varying room reverberation and background noise conditions were used for the assessment. The assessment revealed significant talker gender effects, both with and without face masks. Gender interactions were observed, with girls demonstrating significant differences in speech recognition scores based on talker gender, whereas boys showed no such variations. Face masks attenuated the talker gender effect on speech recognition. Listener gender showed no significant impact in the overall analysis; however, thicker face masks were associated with improved speech recognition at lower reverberation times and noise levels. Reverberation time significantly affected speech recognition only in younger children (mean age: 74 months). Face masks reduced vowel working space areas across both genders. Thus, optimising the acoustic environment is crucial for younger children wearing face masks in educational settings. This study has important implications for classroom acoustics and educational spaces during periods of mandatory mask usage.

## 1 Introduction

Wearing face masks in educational settings has been widely adopted in Korea since the World Health Organisation (WHO) declared an international public health emergency on 30 January 2020 (1). Although wearing a mask outdoors has been voluntary rather than mandatory since 2 May 2022 (2), wearing a mask indoors in schools was made voluntary on 30 January 2023 (3). Additionally, on 11 May 2023, the government’s COVID-19 response headquarters decided to downgrade COVID-19’s status as an emergency (4). According to the revised regulations, the mandatory indoor mask requirement, which previously applied to school buses and tour vehicles, was abolished in June 2023.

The mandatory mask-wearing policy in Korea, which was implemented without a strong lockdown policy (5), has been considered a successful control measure for COVID-19 over the last 3 years. However, it may have affected children undergoing the critical period of language and speech development (6), who have been wearing face masks for over 3 years. Although language development progresses most rapidly until approximately 3.5 years of age (7), continued development during the preschool years remains crucial for phonological mastery (8). Our study examined six year olds who had worn masks for more than two consecutive years (ages 4–6) during this important developmental window. The language capabilities of children at this age serve as a foundation for their academic language skills (9, 10). Therefore, understanding how face masks affect speech recognition in 6 year olds, especially given their extended mask use during preschool, is vital for assessing potential impacts on their linguistic development.

Previous studies have shown that mask-wearing among adults introduces certain challenges in verbal communication and creates a complex acoustic environment that affects sound transmission, language intelligibility, and perceptual comprehension across diverse audiences and environments. Although face masks change the talker’s speech signal, some specific acoustic features, such as voice quality (11), cepstral peak prominence, and vocal intensity (12), are largely unaffected, irrespective of mask type. Face masks are linked to changes in spectral density characteristics that resemble a low-pass filtering effect, thereby reducing the intensity of sounds at higher frequencies, with varying effect sizes (12–14). N95 (US standard) (15) or KN95 (China standard) (16) masks showed a more pronounced impact on speech acoustics than surgical masks (13, 17). The energy ratio between 0 and 1 kHz and 1–8 kHz (LH1000) for sentences significantly increased while wearing either a surgical or KN95 mask (12). The harmonics-to-noise ratios for vowels (18), phrases, and sentences were higher in the mask-wearing conditions than in the no-mask condition (12). KF94 masks (Korea standard comparable to N95 in filtration) (19) demonstrated comparable Speech Transmission Index (STI) attenuation to surgical masks; however, surgical masks exhibited speech level reduction at frequencies of 4 kHz and above, whereas KF94 masks produced attenuation effects at lower frequencies, beginning at 2 kHz (14). The combination of face masks and background noise negatively impacts speech intelligibility for adult listeners (20–22). Badh and Knowles (23), in their scoping review, reported that face masks consistently impact acoustic features of speech, including vocal intensity, measures related to voice quality, and acoustic-phonetic aspects of speech production. Speech intelligibility studies using everyday background noise have documented substantial degradation when speakers wear face masks (24–27).

The impact of face masks on children’s speech recognition is a relevant area of research, with multiple studies examining how different mask types and environmental conditions affect young listeners’ ability to understand spoken language (Table 1). Kwon and Yang (28) found that masks and reverberation times (RT) impaired speech recognition more severely in 4- to 5 year olds than in 6 year olds (*n* = 67) in both quiet and noisy conditions. Children’s speech recognition was significantly impaired by KF94 masks but not by surgical masks. Sfakianaki et al. (24) found that children aged six and seven (*n* = 12) exhibited diminished word recognition in noisy conditions with a surgical mask, despite this mask type having minimal structural interference with speech transmission owing to its loose-fitting design. Lalonde et al. (25) found that the combination of noise and face mask conditions negatively impacted speech understanding in older children aged between 7.4 and 19.8 years with (*n* = 18) and without (*n* = 16) hearing loss. Under auditory-only conditions without visual cues, surgical masks demonstrated optimal performance, with comprehension levels statistically equivalent to unmasked speech. Flaherty et al. (26) also explored the effects of different face masks on word recognition in children aged 8–12 years (*n* = 30) using a two-talker speech masker. They found that the effects depend on the type of face mask being worn. The face shield and transparent mask degraded children’s speech recognition. However, children’s speech recognition in the no-mask condition did not differ from that in either the surgical or N95 masks conditions. All four previous studies (24–26, 28) found that face masks degraded speech recognition in children. However, the effects of each mask type differed (24–26, 28), likely owing to factors such as experimental methods and the age of the target children in limited experimental configurations. Age-related variations in mask effects on speech recognition emerged distinctly across studies. Early childhood research (24, 28) demonstrated developmental differences, with 6 year olds showing better mask tolerance compared to 4- to 5 year olds (*n* = 67) (28), though 6- to 7 year olds (*n* = 12) still exhibited compromised word recognition with surgical masks in noisy conditions (24). Studies of older children (25, 26) revealed different patterns: investigations spanning 7.4–19.8 year old including hard-of-hearing participants (25) and a focused examination of children aged 8–12 years (*n* = 30) (26) demonstrated less mask interference, particularly with surgical masks, suggesting age-dependent improvements in masked speech recognition performance. Studies examining 6-year-old children revealed contrasting findings: Kwon and Yang (28) found that their word recognition scores did not differ significantly between masked and unmasked conditions when they used surgical masks. By contrast, Sfakianaki et al. (24) reported decreased word recognition performance with surgical masks in noisy conditions, although their smaller sample size (*n* = 12) may limit generalisability compared to Kwon and Yang’s (28) larger cohort (*n* = 67). Research focused specifically on 6 year olds is needed to resolve the contradictory findings, particularly given the methodological differences and sample size variations in these studies.

**Table 1:** Experimental conditions: effects of mask type on the speech recognition of children.

Author	Age (years)	Number of children	Mask type	Speech material	Acoustic condition		Talker information
					Noise type (Signal-to-Noise Ratio)	Reverberation time	
Kwon and Yang (28)	4–6	67	No mask Surgical KF94	Korean Standard Monosyllabic Word List for Preschoolers (67)	Babble (SNR 12 dB) No Noise (SNR > 22 dB)	0.6 s 1.2 s	1 Female
Sfakianaki et al. (24)	6–7	2 for pilot & 10 for main	No mask Surgical	Greeklex 2 (68)	Real classroom noise (SNR 2.5 dB) Two-talker noise (SNR 7.5 dB) No Noise	N/A	1 Female
Lalonde et al. (25)	7–18	16 (Normal hearing) & 18 (Bilateral hearing loss)	No mask Surgical (hospital) Fabric Lipview1 (Communicator ™) Lipview2 (ClearMask™)	12 CVs based on the Audiovisual Feature Test for Young Children (69)	Speech-shaped noise (SNR 0 dB)	N/A	1 Female
Flaherty et al. (26)	8–12	30	No mask Surgical N95 Transparent Face shield	30 disyllabic words (70)	Two-talker female speech masker (SNR adaptive)	N/A	1 Female
Present study	6	43	No mask Surgical KF94	Korean Standard Monosyllabic Word List for Preschoolers (67)	Babble (SNR 12 dB) No Noise (SNR > 22 dB)	0.6 s 1.2 s	1 Female & 1 Male

While studies have examined how masks affect speech recognition in children of different ages, another important variable is the gender of the speaker, as it can significantly influence speech intelligibility both with and without masks. Despite its importance, talker gender effects on speech are understudied, with many studies overlooking their possible influences (29). Moreover, studies examining gender differences in speech intelligibility have shown inconsistent findings. Research has demonstrated gender differences in speech intelligibility without masks, gender-specific adaptations to speaking in noisy conditions, and distinct acoustic modification strategies between male and female speakers. Female talkers demonstrated higher intelligibility than other groups of talkers (27, 30–33). In a study of 20 talkers and 200 listeners, Bradlow et al. (30) constructed the profile of a highly intelligible talker as female, producing sentences with a relatively wide range in fundamental frequency (F0), employing a relatively expanded vowel space that covers a broad range in F1, precisely articulating her point vowels, and demonstrating high precision in inter-segmental timing. Markham and Hazan (31) tested intelligibility using a real-word open-set perception test with four talker groups (18 women, 15 men, 6 girls, and 6 boys) and three listener groups (45 adults, 45 children aged 11–12, and 45 children aged 7–8). They also noted that female talkers exhibited higher intelligibility, which led them to conclude that talker intelligibility is primarily influenced by factors inherent to the talker rather than by a combination of factors involving both the talker and listener. Ferguson (32) tested vowel intelligibility in clear and conversational speech with four talker groups (11, 18–24 years; 10, 25–31 years; 10, 32–38 years; and 10, 39–45 years) and found that female talkers performed better in clear speech vowel intelligibility compared to male talkers. In a study of 20 male and 20 female talkers, Kwon (33) found that women exhibited significantly higher speech intelligibility scores than men, with significant differences between men and women in most acoustic parameters: F0, F0 range, formant frequency, formant ranges, vowel working space area, and vowel dispersion. Kwon and Yang (27) investigated the effects of face masks and gender on speech recognition for university students and found that the male speaker exhibited significantly degraded speech recognition with a signal-to-noise ratio of 0 dB. These documented acoustic and phonetic differences suggest that masks could differentially affect speech transmission and recognition based on talker gender.

Although these studies have demonstrated female talkers’ superior intelligibility in certain contexts, other studies have presented contrasting findings, particularly in conversational settings (34–36). Gengel and Kupperman (34) tested word discrimination in noise with 42 college students using three female and three male talkers. They found that the rank order of speaker intelligibility scores was not related to the speaker’s gender and therefore did not account for the differences found. Bradlow and Bent (35) used the Revised Bamford-Kowal-Bench Standard Sentence Test with two talkers and 64 adult listeners and found no talker gender effect for conversational speech or clear speech. Bradlow et al. (36) investigated speech-in-noise perception abilities in children with (*n* = 63) and without (*n* = 36) learning disabilities, using both male and female talkers. They also reported that listeners perceived both talkers to have equivalent baseline conversational speech intelligibility. By contrast, the clear speech intelligibility of the female talker was significantly higher than that of the male talker, leading to an overall greater clear speech benefit observed for the female talker than the male talker. Ferguson and Morgan (37) investigated talker differences in clear and conversational speech in young adults with normal hearing and in hard-of-hearing older adults. They also showed that women received significantly higher ratings than men for clear speech but not for conversational speech. Moreover, the gender difference was noticeably greater for young normal-hearing listeners than for hard-of-hearing older listeners.

The present study investigated how face masks affect speech recognition in 6-year-old Korean children—an age that marks a critical transition in language development and early academic learning. We examined differences between male and female talkers using auralised classroom acoustics to reflect real-world conditions. This study addresses a gap in understanding how talker gender influences masked speech perception during this crucial developmental stage, which is particularly relevant given the prolonged mask use.

## 2 Methods

### 2.1 Listeners

Forty-three 6-year-old children (19 boys and 24 girls) participated in the study with parental consent. The informed consent procedure was approved by the Institutional Review Board of Gwangju University. All the children were monolingual Korean speakers. Study participants attended preschool during a period of mandatory mask requirements, with daily mask usage exceeding 8 h over more than 24 months in the Gwangju region at the time the study was conducted. We assessed children’s hearing status using questionnaires completed by parents or guardians. Potentially hard-of-hearing participants were excluded based on reports from their parents or guardians. Parents may underestimate their children’s hearing loss, particularly for mild or unilateral deficits and those primarily affecting non-speech frequencies (38, 39). However, parents can often detect speech-related hearing issues without formal testing (39). The Receptive and Expressive Vocabulary Test (REVT) (40, 41) for Koreans was used to evaluate children’s language development. By confirming age-appropriate vocabulary skills through REVT, we could attribute differences in word recognition performance to the experimental conditions (masks, reverberation time, talker gender) rather than to underlying language development variations. We excluded data from five children with developmental language delay from the analysis. The speech recognition test and REVT were conducted between 24 February 2022 and 26 April 2022.

We divided the children into two groups: 72–78 months (younger) and 78–84 months (older). 6-year-old children are in a transition period between preschool and school age. Children aged 72–78 months are in a crucial stage for subsequent or concurrent literacy acquisition (42). The grouping could be meaningful at 6-month intervals for 1 year through 84 months, considering children’s language development (43, 44). Table 2 shows the descriptions of the participants, excluding children with developmental language delay. The participants’ mean age was 77 months. The mean ages of the younger and older groups were 74 and 82 months, respectively.

**Table 2:** Number of participants (REVT: normal) and mean age (in months).

Normal	Gender	Girls		Boys		Total	
Total	Number	21		17		38	
	Mean (months)	76.9		76.5		76.7	
	SD	4.86		4.26		4.54	
Sub		72 ≤ Age <78 m	78 ≤ Age <84 m	72 ≤ Age <78 m	78 ≤ Age <84 m	72 ≤ Age <78 m	78 ≤ Age <84 m
	Number	13	8	11	6	24	14
	Mean (months)	73.5	82.4	73.8	81.3	73.6	81.9
	SD	2.40	1.19	2.32	1.86	2.32	1.54

### 2.2 Talkers and speech recordings while wearing face masks

In this study, a professional 51-year-old male voice actor and a 47-year-old female voice actor with more than 20 years of experience participated as speakers.

For the speech recognition test, we used the Korean Standard Monosyllabic Word List for Preschoolers (KS-MWL-P) (45), developed in accordance with the international standard for speech audiometry (46) and word intelligibility by picture identification test (47). Each KS-MWL-P consists of four 25-word lists.

**Figure 1:**
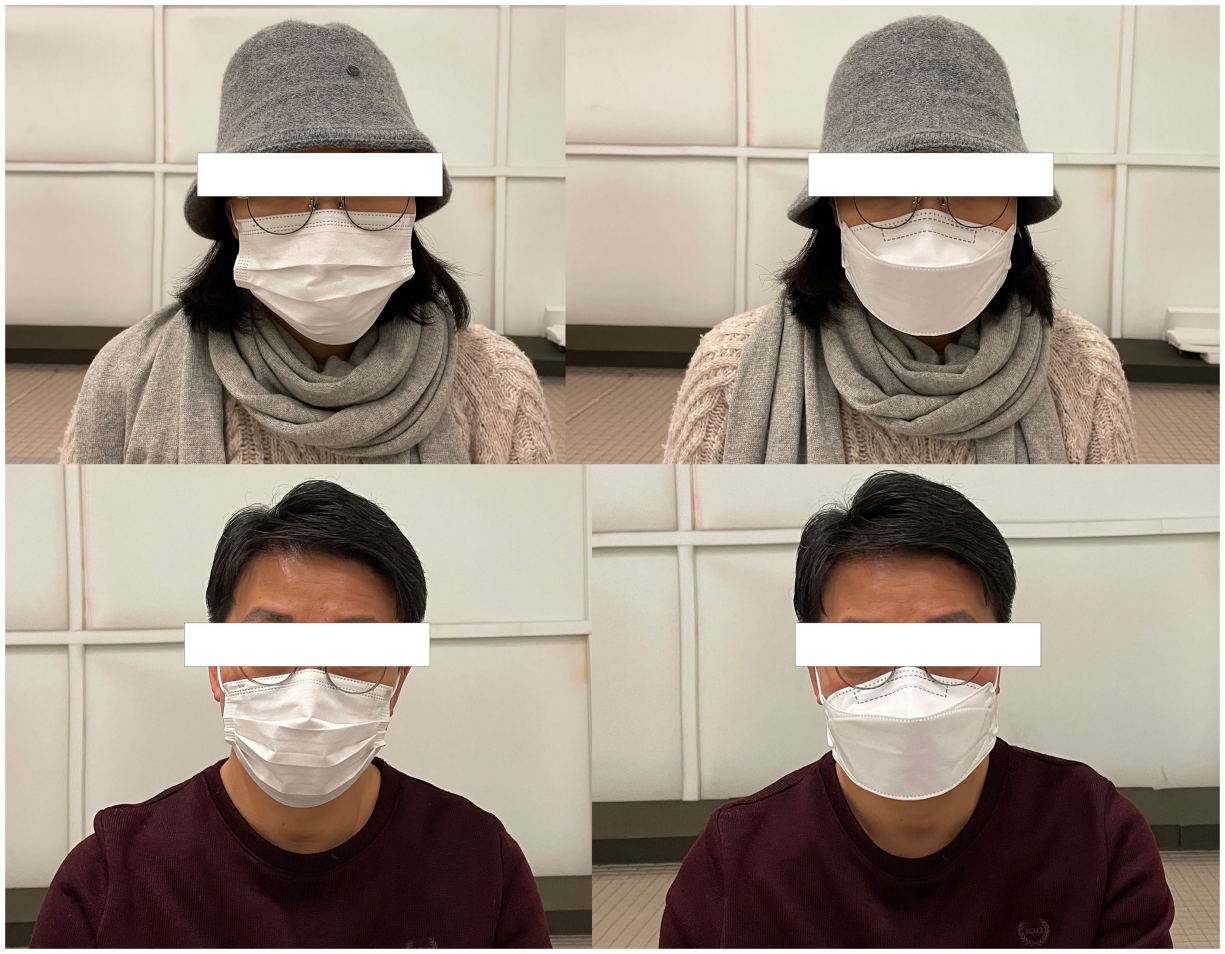
Face masks used in the study (left: surgical, right: KF94).

The KS-MWL-P and the three vowels (/a/, /i/, and /u/) were recorded by the talkers with and without face masks in a fully anechoic chamber (48) ( $f_{g}$ = 50 Hz, 7.0 m × 8.2 m × 7.5 m). The recording was made using the Class 1 sound level metre (Rion NL-52) (49) and analysed using Praat version 6.3.03 (50). Surgical and KF94 masks, two widely utilised face coverings during the COVID-19 pandemic, were selected as the experimental conditions (51), as shown in Figure 1. A surgical mask (thickness: 0.40 ± 0.02 mm) is a disposable face covering that creates a protective barrier between the wearer’s mouth and nose and surrounding contaminants. Although its loose fit allows comfortable breathing, this design provides only partial protection against airborne particles (52). The mask’s breathability makes it a popular choice for daily use despite its limitations (53). The KF94 mask (thickness: 0.61 ± 0.02 mm) is a type of respirator that conforms to the Korean filter standard and is considered equivalent to the N95 mask. The ‘94’ in KF94 refers to its filtration efficiency, indicating that it can filter out at least 94% of particles (54). Speech recording was also conducted in the absence of face masks to serve as a baseline control condition.

**Figure 2:**
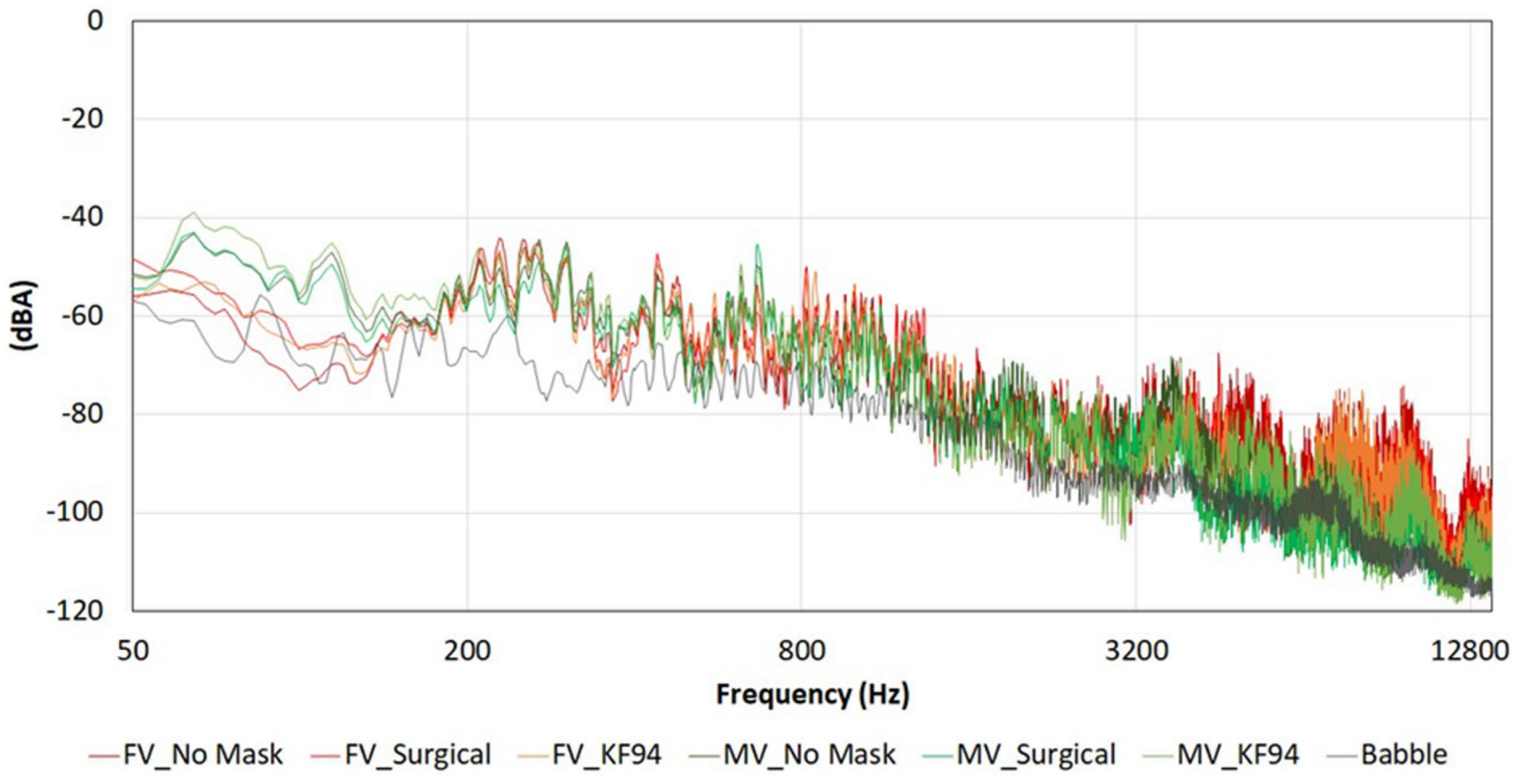
Frequency spectra of talkers with and without face masks, and noise.

The voice actors were asked to speak naturally during the recording, as they would normally speak when wearing a face mask. Figure 2 shows the frequency spectra of the recorded speech sources and babbling noise. We performed phonetic analysis using Praat version 6.3.03 (50).

### 2.3 Classroom acoustical simulation and auralisation

**Figure 3:**
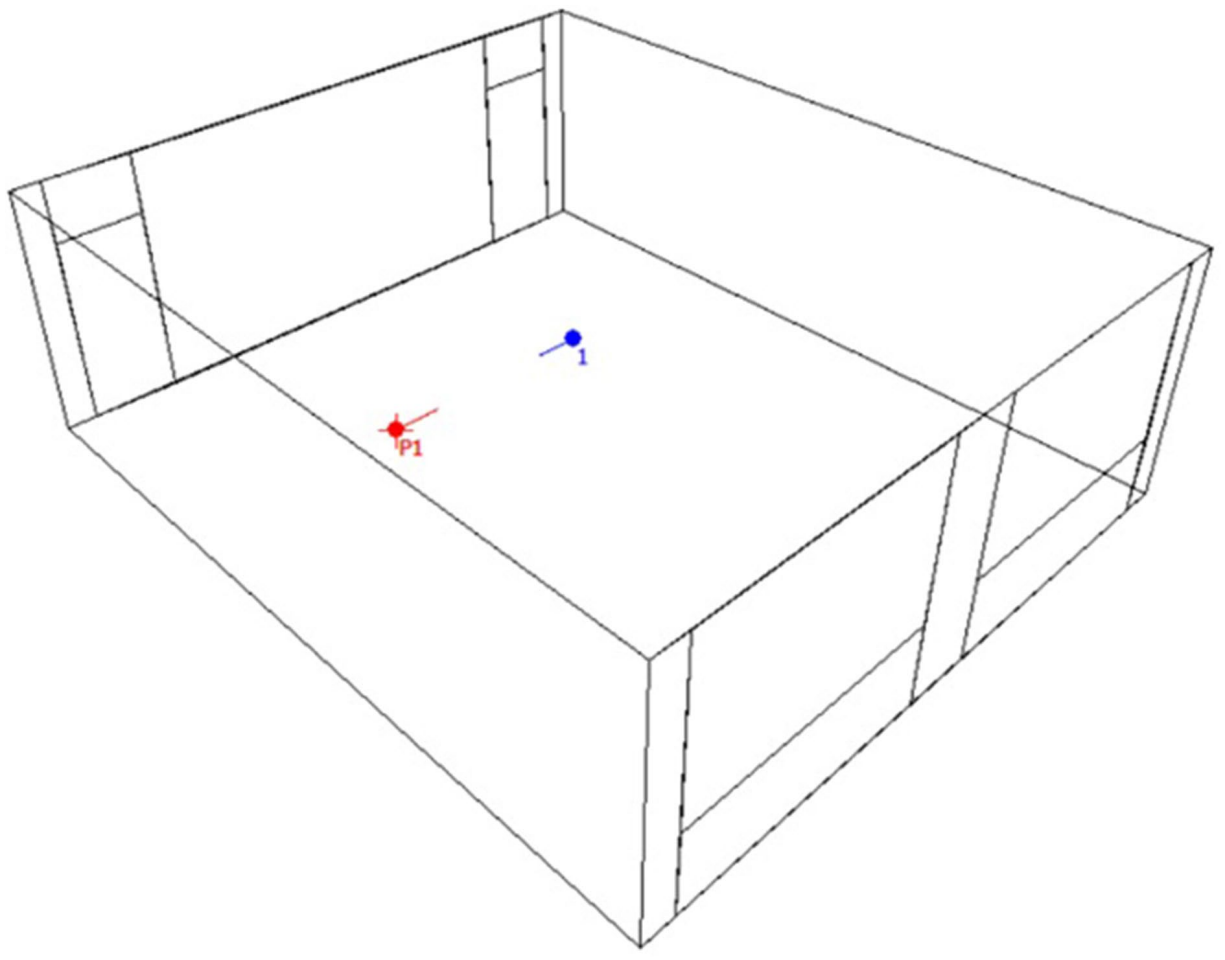
An ODEON classroom model with positions for talker (red) and listener(blue).

For simulation purposes, we selected a standard preschool classroom with dimensions of 6.80 m by 8.00 m and a height of 2.64 m as the investigation model (Figure 3). The experimental measurements yielded two distinct RTs at mid-frequencies, with values of 0.6 s and 1.2 s observed at 500 Hz and 1 kHz, respectively (Table 3). These values were achieved at the listener’s position by modifying the surface materials using ODEON 15.16 (55). The absorption and scattering coefficients of the materials used to achieve each RT were consistent with those reported in the authors’ previous study (28).

**Table 3:** Octave band reverberation times (RTs).

Case	250 Hz	500 Hz	1 kHz	2 kHz	4 kHz	8 kHz	RT_500, 1k_
0.6 s	0.55	0.60	0.58	0.58	0.55	0.43	0.55
1.2 s	0.82	1.16	1.33	1.46	1.52	0.89	1.20

After the reverberation in the simulation models was adjusted, anechoic speech recordings were played back in the two simulated classrooms with RTs of 0.6 and 1.2 s, respectively. We modelled the speech source with the directivity characteristics of a human speaker using ODEON 15.16 (BB93. RAISED NATURAL. SO8) (56). Headphone transfer functions of the Sennheiser HD600 and head-related transfer functions of the KEMAR were applied during the auralisation process. We conducted speech testing under two noise conditions: with the speech source alone (no background noise) and with background noise. The speech source level was calibrated at 62 dBA, whereas the simulated classroom babble at 50 dBA served as the ambient noise. Consequently, the signal-to-noise ratios (SNRs) included both 12 dB and values greater than 22 dB.

### 2.4 Speech recognition test design and procedure

We designed 24 experimental configurations (two talker genders × three mask conditions × two RTs × two noise conditions) and conducted the tests between February and March 2022 in a dedicated, quiet classroom specifically allocated for testing in a preschool. The test procedure was identical to that described in the authors’ previous study (28). We randomly selected 10 words from the KS-MWL-P for each experimental setup using a custom programme developed for this study. These words were presented on a tablet, which allowed the children to respond. The children listened to the words using Sennheiser HD 600 headphones and selected a picture among six pictures on the tablet based on what they heard. Their responses were automatically saved in a database. The children were tested individually at their assigned desks in a quiet classroom specifically designated for testing. Each participant performed the full test with 24 configurations divided into four to six sessions, depending on their level of concentration. Each session lasted less than 5 min. We calculated speech recognition scores as the number of correctly identified words out of 10 per experimental condition and automatically recorded them in the database.

Following the Anderson-Darling normality test, the data were not normally distributed and were analysed using a non-parametric statistical approach with Minitab® 21.1 (57). We employed the Mann–Whitney U and Kruskal-Wallis tests to assess the impact of face masks on speech recognition, with statistical significance set at *p* < 0.05. We conducted statistical power analyses using G*Power’s (58) exact calculations for nonparametric tests. The analyses focused on main effects and selected pairwise comparisons, which demonstrated adequate statistical power with statistically significant results (*p* < 0.05).

## 3 Results

### 3.1 Phonetic acoustic analysis

The observational fundamental frequencies (F0) of the two speakers are listed in Table 4. The mean fundamental frequencies of the three vowels were determined by analysing three separate recordings of each vowel. We calculated the average F0 by first measuring the F0 of each list from the four KS-MWL-P lists, and then taking the mean of these measurements. The mean F0 was 103.39 Hz for the male voice actor and 240.00 Hz for the female voice actor. The male talker’s mean F0 was lower than the average male voice F0 of 120 Hz, whereas the female talker’s F0 was higher than the average female voice F0 of 230 Hz (59). In general, the F0s of men are lower than those of women, which is consistent with established literature on gender differences in vocal acoustics. Regarding the effect of face masks, we observed a gender-dependent pattern: the male talker showed a decrease in F0 when wearing masks, whereas the female talker demonstrated an increase in F0. Although there were changes in F0 according to the presence or absence of masks, in this study, the changes in F0 values according to mask thickness were not clearly evident. This finding represents an observed trend in our specific talkers rather than a robust effect generalisable across speakers. For the male talker, a consistent negative relationship was observed between mask thickness and F0. The baseline condition (no mask) exhibited the highest mean frequency at 103.39 Hz, followed by a decrease to 97.00 Hz with the surgical mask and a further reduction to 94.00 Hz with the KF94 mask, representing the lowest frequency across all conditions. By contrast, the female talker demonstrated an inconsistent pattern. Whereas the surgical mask condition showed an expected decrease in F0 from the baseline (240.00 Hz–211.00 Hz), the KF94 mask condition unexpectedly exhibited a higher frequency (229.00 Hz) compared to the surgical mask condition.

**Table 4:** Fundamental frequencies (F0s) of male and female voices.

Mask	Fund. Freq (Hz)	Male voice		Female voice	
		Mean	*SD*	Mean	*SD*
No mask	/a/	79.91	9.07	200.95	6.40
	/i/	101.49	4.97	227.29	8.76
	/u/	93.60	9.62	191.18	28.87
	MWL	103.39		240.00	
Surgical mask	/a/	85.83	8.33	210.62	16.88
	/i/	92.03	10.61	235.74	5.53
	/u/	71.30	8.28	215.54	47.13
	MWL	97.00		211.00	
KF94 mask	/a/	80.45	1.40	218.14	4.04
	/i/	59.33	4.32	231.35	3.90
	/u/	74.87	4.04	209.95	34.11
	MWL	94.00		229.00	

MWL, Monosyllabic word list from KS-MWL-P.

**Figure 4:**
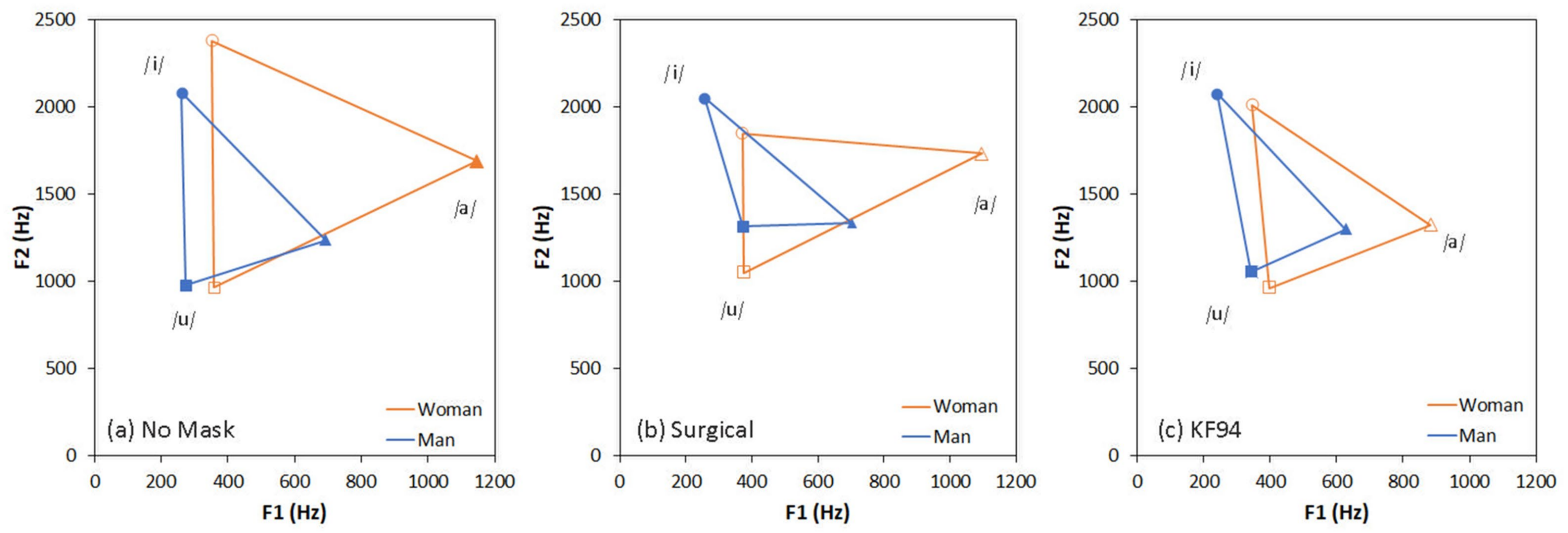
Vowel working space area (VWSA) for each mask-wearing condition.

The formant frequencies and vowel working space areas of the three vowels in three mask conditions are listed in Table 5. The face mask reduced the vowel working space area regardless of gender (Figure 4). In the formant analysis of the /a/ sound, F1 frequencies were higher than those of /i/ and /u/ for both the male talker and the female talker. For the /u/ sound, the difference in F1 and F2 frequencies between the male talker and female talker was relatively smaller than that for /a/ or /i/. The difference between F2 and F1 (F2 − F1) for both genders was less significant than that between vowels. The female talker’s vowel working space area without a face mask was twice that of her male counterpart.

**Table 5:** Comparisons of F1, F2, and F2 − F1 and vowel working space area.

Mask	Formant	Talker gender	/a/		/i/		/u/		VWSA (Hz^2^)	
			Mean	*SD*	Mean	*SD*	Mean	*SD*	Male voice	Female voice
No mask	F1	M	693.68	56.70	260.59	18.08	274.52	65.92	233,764.2	559,504.3
		F	1146.26	111.12	352.98	97.27	358.53	61.17		
	F2	M	1234.42	24.14	2082.79	84.26	975.99	557.29		
		F	1689.04	186.39	2379.41	679.15	963.97	230.89		
	F2 − F1	M	540.74		1822.21		701.47			
		F	542.77		2026.43		605.43			
Surgical mask	F1	M	702.00	209.55	255.74	25.22	373.76	85.29	122,061.4	291,513.2
		F	1096.94	85.92	370.67	178.44	376.09	79.49		
	F2	M	1331.31	313.55	2051.36	50.36	1313.90	842.70		
		F	1733.68	204.53	1850.07	965.64	1046.43	455.26		
	F2 − F1	M	629.31		1795.88		940.14			
		F	636.74		1479.40		670.34			
KF94 mask	F1	M	628.17	147.24	239.85	44.14	342.62	81.17	158,688	247,103.7
		F	882.68	213.53	345.92	52.27	397.70	42.57		
	F2	M	1298.72	228.95	2078.46	50.96	1054.81	681.52		
		F	1322.703	247.12	2012.11	936.89	959.85	133.14		
	F2 − F1	M	670.54		1838.61		712.19			
		F	440.03		1594.58		562.15			

### 3.2 Speech recognition scores

Mann–Whitney U tests were performed. Speech recognition scores were significantly affected by RT, noise, talker gender, and listener age (in months; Table 6). The Kruskal-Wallis test (Tables 7–10) is a non-parametric statistical test that extends the Mann–Whitney U test to compare three or more groups.

**Table 6:** Speech recognition score comparisons (Mann–Whitney U test, *p < 0.05).

Factor	Variable 1	*N*	Median	Variable 2	*N*	Median	*W*-value	*p*-value
Talker Gender	Female voice	446	9	Male voice	449	9	213787.5	< 0.0005*
Listener Gender	Girls	512	9	Boys	383	9	228884.5	0.898
Age (month-old)	72 ≤ Age <78 m	570	9	78 ≤ Age <84 m	325	9	234755.5	< 0.0005*
Mask	No Mask	297	9	Surgical	299	9	90,436	0.397
	Surgical	299	9	KF94	299	9	87369.5	0.302
	KF94	299	9	No Mask	297	9	88,166	0.816
RT	0.6 s	449	9	1.2 s	446	9	209076.5	0.040*
Noise	SNR > 22 dB	441	9	SNR = 12 dB	454	9	194376.5	0.020*

**Table 7:** Speech recognition scores by face mask type (Kruskal-Wallis test, *p < 0.05).

Factor	Variable	Face mask type											
		No mask				Surgical mask				KF94 mask			
		N	Median	Mean rank	Z-value	N	Median	Mean rank	Z-value	N	Median	Mean rank	Z-value
Talker Gender	Female	148	9	162.0	2.59	149	9	164.9	2.96	149	9	153.9	0.78
	Male	149	9	136.1	−2.59	159	9	135.2	−2.96	150	9	146.1	−0.78
		H	= 6.73	*p*	= 0.009*	H	= 8.79	*p*	= 0.003*	H	= 0.62	*p*	= 0.433
Listener Gender	Girls	171	9	149.8	0.18	170	9	148.6	−0.33	171	9	149.9	−0.02
	Boys	126	9	147.9	−0.18	129	9	151.9	0.33	128	9	150.1	0.02
		H	= 0.03	*p*	= 0.854	H	= 0.11	*p*	= 0.740	H	= 0.00	*p*	= 0.983
Age	72 ≤ Age <78 m	189	9	135.6	3.35	190	9	133.4	−4.39	191	9	143.6	−1.70
	78 ≤ Age <84 m	108	9	172.4	3.35	109	9	179.0	4.39	108	9	161.3	1.70
		H	= 12.61	*p*	< 0.0005*	H	= 19.25	*p*	< 0.0005*	H	= 2.89	*p*	= 0.089
RT	0.6 s	150	9	154.3	1.07	149	9	152.9	0.57	150	9	159.3	1.86
	1.2 s	147	9	143.6	−1.07	150	9	147.2	−0.57	149	9	140.7	−1.86
		H	= 1.14	*p*	= 0.285	H	= 0.33	*p*	= 0.568	H	= 3.46	*p*	= 0.063
Noise	SNR > 22 dB	146	9	150.5	0.29	148	9	159.0	1.79	147	9	159.7	1.92
	SNR = 12 dB	151	9	147.6	−0.29	151	9	141.2	−1.78	152	9	140.6	−1.92
		H	= 0.09	*p*	= 0.769	H	= 3.16	*p*	= 0.076	H	= 3.67	*p*	= 0.055

**Table 8:** Speech recognition scores by classroom acoustics (Kruskal-Wallis test, * *p* < 0.05).

Factor	Variable	Reverberation time (RT) x Noise conditions															
		RT0.6 s No Noise (SNR > 22 dB)				RT0.6 s Babble (SNR = 12 dB)				RT1.2 s No Noise (SNR > 22 dB)				RT1.2 s Babble (SNR = 12 dB)			
		N	Median	Mean rank	Z-value	N	Median	Mean rank	Z-value	N	Median	Mean rank	Z-value	N	Median	Mean rank	Z-value
Talker Gender	Female	108	9	118.2	2.03	114	9	130.1	3.16	111	9	117.2	1.19	113	9	115.9	0.91
	Male	110	9	100.9	−2.03	117	9	102.3	−3.16	112	9	106.9	−1.19	110	9	108.0	−0.91
		H	= 4.10	*p =*	0.043*	H	= 10.00	*p* =	0.002*	H	= 1.41	*p* =	0.234	H	= 0.84	*p* =	0.360
Listener Gender	Girls	122	9	113.9	1.17	135	9	114.6	−0.38	127	9	110.1	−0.51	128	9	110.5	−0.40
	Boys	96	9	103.8	−1.17	96	9	118.0	0.38	96	9	114.5	0.51	95	9	114.0	0.40
		H	= 1.38	*p* =	0.241	H	= 0.14	*p* =	0.706	H	= 0.26	*p* =	0.609	H	= 0.16	*p* =	0.690
Age	72 ≤ Age <78 m	140	9	99.6	−3.11	146	9	110.4	−1.68	145	9	102.7	−2.94	139	9	100.3	−3.47
	78 ≤ Age <84 m	78	9	127.3	3.11	85	9	125.7	1.68	78	9	129.3	2.94	84	9	131.3	3.47
		H	= 9.69	*p =*	0.002*	H	= 2.83	*p* =	0.093	H	= 8.63	*p* =	0.003*	H	= 12.05	*p* =	0.001*
Mask Type	No Mask	72	9	112.1	0.43	78	9	114.9	−0.17	74	9	103.8	−1.33	73	9	123.9	1.93
	Surgical	73	9	101.8	−1.27	76	9	112.5	−0.56	75	9	116.4	0.72	75	9	105.5	−1.07
	KF94	73	9	114.6	0.82	77	9	120.6	0.73	74	9	115.7	0.60	75	9	106.9	−0.84
		H	= 1.68	*p* =	0.432	H	= 0.59	*p* =	0.744	H	= 1.78	*p* =	0.411	H	= 3.73	*p* =	0.155

**Table 9:** Speech recognition scores by children’s age (Kruskal-Wallis test, *p < 0.05).

Factor	Variable	Age							
		72 ≤ Age <78 m				78 ≤ Age <84 m			
		N	Median	Mean rank	Z-value	N	Median	Mean rank	Z-value
Talker Gender	Female	282	9	298.9	1.93	164	9	178.9	3.07
	Male	288	9	272.3	−1.93	161	9	146.9	−3.07
		H	= 3.72	*p*	= 0.054	H	= 9.42	*p*	= 0.002*
Listener Gender	Girls	330	9	285.3	−0.04	182	10	165.9	0.62
	Boys	240	9	285.8	0.04	143	9	159.3	−0.62
		H	= 0.00	*p*	= 0.970	H	= 0.39	*p*	= 0.534
Mask Type	No Mask	189	9	286.9	0.15	108	9	165.5	0.34
	Surgical	190	9	268.1	−1.79	109	9	166.8	0.52
	KF94	191	9	301.4	1.64	108	9	156.7	−0.86
		H	= 3.93	*p*	= 0.140	H	= 0.75	*p*	= 0.688
RT	0.6 s	286	9	300.2	2.14	163	9	166.2	0.61
	1.2 s	284	9	270.7	−2.14	162	9	159.8	−0.61
		H	= 4.58	*p*	= 0.032*	H	= 0.38	*p*	= 0.539
Noise	SNR > 22 dB	285	9	296.9	1.65	156	9	173.5	1.93
	SNR = 12 dB	285	9	274.1	−1.65	169	10	153.3	−1.93
		H	= 2.74	*p*	= 0.098	H	= 3.74	*p*	= 0.053

**Table 10:** Speech recognition scores by listener gender (Kruskal-Wallis test, *p < 0.05).

Factor	Variable	Listener gender							
		Girls				Boys			
		N	Median	Mean rank	Z-value	N	Median	Mean rank	Z-value
Talker Gender (No Mask Only)	Female	85	9	96.5	2.75	63	9	65.8	0.69
	Male	86	9	75.5	−2.75	63	9	61.2	−0.69
		H	= 7.59	*p*	= 0.006*	H	= 0.48	*p*	= 0.488
Talker Gender (All)	Female	255	9	279.7	3.54	191	9	200.0	1.41
	Male	257	9	233.5	−3.54	192	9	184.0	−1.41
		H	= 12.52	*p*	< 0.0005*	H	= 1.99	*p*	= 0.158

#### 3.2.1 Effects of talker gender and face masks

We analysed the effects of face masks on speech recognition using Kruskal-Wallis tests across multiple factors (Table 7). In terms of gender, female talkers consistently achieved higher mean ranks than male talkers in the no-mask (162.0 vs. 136.1, *p* = 0.009) and surgical-mask (164.9 vs. 135.2, *p* = 0.003) conditions, whereas this difference was not significant with KF94 masks (153.9 vs. 146.1, *p* = 0.433). Listener gender showed no significant differences across all mask conditions (*p* > 0.05). However, age demonstrated highly significant effects in both no-mask and surgical-mask conditions (*p* < 0.0005), with older children performing better than younger children. This age-related difference was not significant in the KF94 mask condition (*p* = 0.089). Response-time conditions (0.6 s vs. 1.2 s) showed no significant differences across all mask types (*p* > 0.05). Similarly, noise conditions (SNR > 22 dB vs. SNR = 12 dB) did not significantly affect speech recognition scores in any mask condition, although we observed a marginal trend towards significance in the KF94 mask condition (*p* = 0.055).

#### 3.2.2 Effects of talker gender and classroom acoustics

We analysed speech recognition performance across various acoustic conditions, combining RT and noise levels (Table 8). Female talkers showed significantly higher mean ranks than male talkers in quiet conditions with short RT (0.6 s, SNR > 22 dB: 118.2 vs. 100.9, *p* = 0.043) and in babble noise conditions with short RT (0.6 s, SNR = 12 dB: 130.1 vs. 102.3, *p* = 0.002). However, this gender difference disappeared in conditions with longer RT (1.2 s), regardless of noise level (*p* > 0.05). Listener gender showed no significant differences across all acoustic conditions (*p* > 0.05). Age effects were significant in three out of four conditions, with older children consistently performing better than younger children in quiet conditions with both short and long RT (0.6 s: *p* = 0.002; 1.2 s: *p* = 0.003) and in babble noise with long RT (1.2 s: *p* = 0.001). This age-related difference was not significant only in babble noise with short RT (0.6 s: *p* = 0.093). Mask conditions showed no significant effects on speech recognition scores across all acoustic conditions (*p* > 0.05).

#### 3.2.3 Effects of talker gender and children’s age

To examine how different factors affect speech recognition performance across age groups, we analysed multiple acoustic and talker variables separately for younger (72 ≤ Age<78 m) and older (78 ≤ Age<84 m) children (Table 9). In the younger age group, talker gender (female: 298.9 vs. male: 272.3, *p* = 0.054) and RT (0.6 s: 300.2 vs. 1.2 s: 270.7, *p* = 0.032) influenced speech recognition scores, with RT reaching statistical significance. Listener gender, mask type, and noise conditions showed no significant effects (*p* > 0.05). In the older age group, talker gender had a significant effect, with female talkers achieving higher mean ranks than male talkers (178.9 vs. 146.9, *p* = 0.002). We also observed a marginal effect of noise conditions (SNR > 22 dB: 153.3 vs. SNR = 12 dB: 173.5, *p* = 0.053). No significant effects were found for listener gender, mask type, or RT (*p* > 0.05).

#### 3.2.4 Effects of talker gender and listener gender

To understand how listener gender interacts with talker gender, we analysed speech recognition performance separately for girl and boy listeners under different speaker conditions (Table 10). For girl listeners, female talkers achieved significantly higher mean ranks than male talkers both in the no-mask condition (96.5 vs. 75.5, *p* = 0.006) and when all mask conditions were combined (279.7 vs. 233.5, *p* < 0.005). By contrast, boy listeners showed no significant differences in speech recognition scores between female and male talkers, either in the no-mask condition (65.8 vs. 61.2, *p* = 0.488) or when all mask conditions were combined (200.0 vs. 184.0, *p* = 0.158). These results suggest that girl listeners were more sensitive to talker gender differences than boy listeners.

## 4 Discussion

This study investigated the effects of face masks, room acoustics, and talker gender on speech recognition in 6-year-old children. The results showed that face masks lowered the fundamental and formant frequencies for both male talker and female talker. The talker gender effect was driven by the girls’ performance and was not consistent across all listeners. Female speakers had higher speech recognition scores among female children and older children, although this advantage diminished with KF94 masks and longer RTs (1.2 s).

### 4.1 Effect of talker gender on speech recognition: adult talker and 6-year-old listener

The analysis revealed distinct patterns in how talker gender affected speech recognition among children. Whereas Table 6 shows a significant overall effect favouring female voices, Table 10 demonstrates that this effect was not uniform across listener groups. Specifically, girls showed significantly better performance with female talkers than with male talkers, whereas boys showed no significant difference in their performance between female and male talkers. This indicates that the overall talker gender effect observed in the aggregate data stemmed primarily from the girls’ enhanced performance with female voices, rather than reflecting a pattern common to all children. This finding provides an important context for interpreting the talker gender effect on speech recognition. Rather than being a universal phenomenon, the influence of talker gender appears to be specific to female listeners. The term ‘listener gender effect’ may therefore require refinement as the data suggest a more specific interaction between talker and listener gender, primarily manifesting in female listeners’ response to female voices. This is consistent with previous studies (30–33) reporting that women’s speech was more intelligible than men’s speech both with and without face masks. Conversely, a more detailed analysis based on listener gender revealed that talker gender did not have an effect on boys’ speech recognition, which is consistent with Yoho et al.’s (29) study on adult listeners. The rank orders of the girls’ speech recognition scores with the female voice were significantly higher than those with the male voice. However, the rank orders of the boys’ speech recognition scores did not differ significantly by talker gender, with or without face masks.

Studies on talker gender perception have revealed several key findings across physiological, acoustic, and perceptual domains. Sex-specific differences in vocal tract dimensions significantly affect articulation and vowel space characteristics (60). In terms of intelligibility, male speakers showed slightly higher intelligibility than female speakers, potentially owing to differences in speech harmonics or systematic variations in consonant articulation (61). Brain imaging studies revealed gender-specific neural networks involved in voice processing, particularly in areas associated with auditory processing and attention (62). Perceptual studies demonstrated that listeners generally showed greater accuracy in classifying voices of the opposite gender, which suggests that voice processing is influenced by both the listener’s sex and sexual orientation (63). Additionally, studies on male voices found that less phonetically distinct speech was perceived as more masculine, highlighting the complex relationship between acoustic features and gender perception (64).

Neither studies on the talker gender effect on speech recognition nor those on the acoustic or phonetic causes of the talker gender effect on speech recognition have comprehensively identified sex-dimorphic acoustic differences. Further research is required to determine the factors underlying these differences.

### 4.2 Face mask and acoustical aspects on speech recognition in 6-year-old children

The present investigation corroborates and extends the existing literature on the influence of face masks on speech perception and recognition. The acoustic effects observed, particularly the constriction of vowel working space areas, are consistent with previously documented findings (18, 65). However, speech recognition patterns among 6 year olds were more complex than previously documented. Although a previous study (28) found no statistical changes in speech recognition among 6 year olds when using KS-MWL-P materials with face masks, our analysis revealed substantial variability within the 6-year-old group when stratified by 6-month intervals. Specifically, 6-month interval differences emerged in conditions with no masks or with surgical masks, but not with KF94 masks.

The heightened sensitivity to RT observed in younger participants corroborates the findings from our previous study (28) on 4- and 5-year old participants, thereby strengthening the empirical foundation of age-dependent acoustic sensitivity. Additionally, our observation of talker gender effects exclusively in older children constitutes a previously undocumented phenomenon that merits further empirical investigation. These results support and augment the recommendations made by Sfakianaki et al. (24) regarding acoustically optimised learning environments, particularly given the observed relationship between mask utilisation and children’s acoustic environmental preferences: relatively short RTs and low noise levels. Controlling reverberation is particularly crucial for younger children’s speech comprehension. However, no significant difference in performance between different RTs (*p* = 0.539) was observed among older children, which indicates more resilience to varying acoustic conditions. The interaction between reverberation and mask conditions provides additional design considerations. Under each mask condition, RT showed no significant effect on speech recognition. However, the trend towards significance with KF94 masks (*p* = 0.063) suggests that acoustic optimisation may become more critical when face masks are used in learning spaces. These findings indicate that acoustic design should prioritise shorter RTs (0.6 s), particularly in learning spaces for younger children. The results also suggest that acoustic optimisation becomes increasingly important when additional speech barriers, such as face masks, are present in the learning environment during the developmental transition from preschool to elementary school.

### 4.3 Limitations and future works

First, only one male and one female speaker participated in the speech recording. Although they cannot represent all male and all female voices, the phonetic differences in the F0s between the two talkers were valid as distinct gendered voices. The male talker’s F0 was lower than that of the average male, whereas the female talker’s F0 was higher than that of the average female (59). Although talker gender effects have been reported in the literature, extensive variability across talkers within a given gender has also been reported. Given the specific talkers selected, whether the results can be generalised to the larger population or attributed to the specific talkers selected for this study, regardless of gender, may not be clear. Including more talkers in future work would help confirm whether the effects found here are consistent across voices and would strengthen conclusions about gender-related speech recognition patterns.

Second, the 6-year-old children, who had just finished their preschool programme and would enter elementary school, showed ceiling effects in their speech recognition tests. These ceiling effects likely resulted from a combination of factors including the signal-to-noise ratio (SNR) used, stimulus difficulty calibration, and possibly the closed-set test format. While the closed-set format was developmentally appropriate for this age group, future studies should consider adjusting SNR levels or implementing more challenging stimulus materials to better capture performance variability. Although some studies have successfully conducted speech recognition tasks with 6- or 7 year olds using various methodologies (31, 66), careful consideration of both test format and stimulus difficulty is needed to address potential ceiling effects while accommodating individual differences in language development.

Third, in this study with a limited sample of 6-year-old children, the influence of talker gender appeared to be specific to girls. However, these findings should be interpreted cautiously given the small sample size, and further research with larger, more diverse groups are needed to establish whether this pattern generalises to broader populations of young children.

Fourth, the speech materials were restricted to clear laboratory speech with monosyllabic words; however, we attempted to simulate the acoustic qualities of a realistic listening environment, such as room reverberance and background noise. Future studies should consider using real-life speech materials for children.

Finally, an important limitation is that all participants were monolingual Korean speakers. This linguistic homogeneity may limit the generalizability of findings to children from other language backgrounds. Korean has distinct phonetic and prosodic characteristics that could influence how children process masked speech and respond to talker gender cues. The language’s specific vowel system, consonant inventory, and prosodic patterns differ substantially from other languages, potentially affecting both the acoustic impact of face masks and the salience of gender-related vocal cues. Additionally, cultural factors related to voice perception and gender identification may vary across different linguistic communities. Future cross-linguistic studies would be valuable to determine whether the observed patterns—particularly the gender-specific effects in girls’ speech recognition with female talkers—hold across diverse language backgrounds and cultural contexts.

## 5 Conclusion

Our study revealed that talker gender effects on speech recognition are highly specific rather than universal. Female listeners (girls) demonstrated significant differences in speech recognition based on talker gender, while male listeners (boys) showed no such effect. This clarifies that the interaction between talker gender and listener gender was asymmetrical, with effects primarily observed in one listener group. Age-related effects were similarly condition-specific: talker gender influenced speech recognition only among older 6-year-old children (average age: 82 months), with no significant effect detected among younger 6 year olds (average age: 74 months).

The impact of face masks on talker gender perception varied distinctly by mask type. Surgical masks partially preserved talker gender effects, whereas KF94 masks eliminated these differences. This finding demonstrates that mask effects are not uniform but depend specifically on mask properties. We emphasise that listener gender alone did not produce significant main effects in our overall analysis; effects emerged only through specific interactions with other variables.

Under more challenging listening conditions—specifically when talkers wore KF94 masks or in environments with longer reverberation times—no talker gender effects were observed. Thicker face masks performed optimally only in specific acoustic environments characterised by shorter reverberation times and lower background noise levels. Reverberation time in classrooms particularly affected the youngest children in our study (average age: 74 months), indicating that acoustic environment optimisation should be age-targeted.

The acoustic analysis confirmed that face masks reduced the vowel working space area for both male and female talkers, directly diminishing acoustic clarity. This finding has a direct practical implication: classrooms where masked speech is common should be specifically designed with shorter reverberation times and minimised background noise to compensate for the reduced spectral information available to younger listeners processing masked speech.

## Data Availability

The raw data supporting the conclusions of this article will be made available by the authors, without undue reservation.
